# ‘Family Violence and Football’ at 15: Revisiting the Effect of Emotional Cues on Intimate Partner Violence

**DOI:** 10.3390/ijerph23060768

**Published:** 2026-06-07

**Authors:** Clay Collins

**Affiliations:** Department of Kinesiology, University of Georgia, 330 River Rd, Athens, GA 30602, USA; coll6968@uga.edu

**Keywords:** intimate partner violence, emotional cues, National Football League

## Abstract

**Highlights:**

**Public health relevance—How does this work relate to a public health issue?**

- Intimate partner violence (IPV) is a major health issue.
- Understanding the determinants of IPV is vital to designing elimination strategies.

**Public health significance—Why is this work of significance to public health?**

- This work examines in further detail the role of unexpected, seemingly irrelevant events (“emotional cues”) on IPV occurrence.
- This work updates a seminal paper on the topic and finds that the role emotional cues have on IPV has changed over time.

**Public health implications—What are the key implications or messages for practitioners, policy makers and/or researchers in public health?**

- IPV can be subject to even the smallest triggers, and understanding what types of events can serve as triggers can help design IPV prevention strategies.

**Abstract:**

This paper revisits the famous Card and Dahl study on emotional cues on intimate partner violence (IPV). This paper was one of the first to explore the effects of seemingly irrelevant outcomes—in this case, local National Football League (NFL) game outcomes—on incidents of IPV, finding that upset NFL losses are associated with a 10% increase in IPV. This paper briefly describes the current state of the “emotional cue” literature, specifically further extensions related to IPV, and then applies the Card–Dahl model to an updated 25-year crime dataset. Results suggest a deviation from the previous literature. NFL upset losses seem to have a considerably reduced effect on IPV than initially found, but some restricted samples find relationships between unexpected game outcomes and IPV.

## 1. Introduction

Intimate partner violence (IPV) is a major problem both in the United States and worldwide. Kudon et al. [1] estimate that 29.9% of U.S. women experience at least one IPV-related impact in their lifetime (which includes consequences such as being physically injured, missing at least one day of work or school, filing a police report, needing medical attention, and/or being concerned for one’s safety). As a result, studies of the potential mechanisms of IPV are widespread. Though a consensus on the exact mechanism behind IPV has not been reached, one of the most popular theories is that IPV arise unintentionally when a relationship argument escalates out of control. Proposed by Straus et al. [2], this mechanism has been of chief interest to researchers as the source of a theoretical framework for the sources of IPV. As formulated mathematically by Card and Dahl [3], this “loss-of-control” model proposes that the probability of IPV breaking out between a couple is dynamic and subject to external influences (“shocks” or “triggers”). Card and Dahl [3] incorporate a behavioral economics framework based upon the concepts of reference-dependent equilibria and loss aversion of Kőszegi and Rabin [4] and Kahneman and Tversky [5], respectively. According to this framework, IPV is most susceptible to change as a result of unexpected triggers. These could be shocks to existing viewpoints or expectations of future outcomes (either relevant or irrelevant).

A wide variety of studies in economics have examined how various triggers can influence (usually increase) the probability of IPV. As summarized by Burke et al. [6], many studies find positive links between IPV and surprise weather shocks (heat, drought, rainfall). Shocks to income also affect IPV probability, with Baranov et al. [7] finding household cash transfers to be associated with decreases in IPV. On the contrary, Amaral et al. [8] and Angelucci and Heath [9] find that employment programs specifically designed for women have the potential to *increase* incidents of IPV. These effects, perhaps, may not be surprising, as considerable household bargaining revolves around income (which in many developing countries is tied to agriculture and therefore affected by weather). However, Card and Dahl [1] find that events completely unrelated to the household itself can play a major role in outbreaks of IPV. The targeted trigger of their study, and the source of considerable follow-up work, is the outcome of sporting events.

Though Card and Dahl [3] is by far the most famous research into the connection between sports and violence, they were not the first. Gantz et al. [10] find a positive relationship between National Football League (NFL) viewership and IPV (regardless of game outcome). In a study of college campuses, Rees and Schnepel [11] find that college football games are associated with spikes in assaults, vandalism, and alcohol-related arrests, with even larger effects found with unexpected outcomes. Upset losses (losses where the home team was favored to win) were associated with the largest spikes in crime. However, Rees and Schnepel [11] do not specifically examine IPV. Furthermore, there is a history of observational and laboratory experiments that find heightened sensations of anxiety and hostility while watching sporting events [12,13,14]. Card and Dahl [1] used observational data from the National Incident-Based Reporting System (NIBRS) to examine the role local NFL upset losses play in IPV. They find that unexpected losses are associated with an approximate 10% increase in IPV cases. Close losses, predicted losses, or upset wins (wins where the local team was expected to lose) had no effect on IPV.

Fifteen years after publication, this paper continues to be a focal point of the so-called “emotional cue” literature and has spawned dozens of extensions. Other IPV spikes have been found using other sports outcomes as possible triggers; some have found mixed results. Examining Scottish soccer, Dickson et al. [15] find no effect of upset losses on IPV, but find large IPV spikes during rivalry games (regardless of match outcome). Cardazzi et al. [16] find a positive relationship between unexpected basketball losses and IPV. Mudrow [17] finds that inaccurate referee decisions (in their case, ball and strike calls by Major League Baseball umpires) can lead to spikes in IPV. Arnesen and Matsuzawa [18] explore a new dimension, the recent legalization of online sports betting in the U.S., finding spikes in NFL upset losses after the state has legalized mobile betting. Subsequent studies have explored other outcome measures beyond IPV, including sexual assaults [19], judicial sentencing [20], retail spending [21], tipping behavior [22], online reviews [23,24], and internet comments [25].

This framework has also been copied and extended to non-sports settings as well, examining how other unexpected events can affect IPV. Beland and Brent [26] find that spikes in traffic, compared to normal levels, can increase IPV incidents in Los Angeles. Collins [27] finds that election results can lead to spikes in IPV—independent of whether the election outcome was predicted or not. Another section of the literature, such as the work of Müller and Schwarz [28,29], explores social media’s role in escalating violence. However, these papers focus on hate crimes, which may or may not follow the “loss-of-control” framework. Other papers have examined unexpected legal or policy changes. Both Durrance et al. [30] and Dave et al. [31] find that restrictions in abortion access in the United States as a result of the *Dobbs* decision have increased IPV incidents. Finally, Leslie and Wilson [32] and Arenas-Arroyo et al. [33] find positive links between COVID-19 lockdowns and IPV rates.

Though this literature is substantial, with Card and Dahl [3] generating over 1000 citations, there are questions regarding the persistence of this effect. Given its impact and the fact that the data used is now over 20 years old, an update of this paper is likely in order. To the author’s knowledge, an update of this paper has not been done before. In addition, like other fields, economics continues to suffer from a “replicability crisis” where results posted in journals may be irreproducible or exaggerated [34]. Replication or updated studies are relatively rare in the economics profession, making an update necessary [35]. The closest replication to Card and Dahl [3] is Arnesen and Matsuzawa [18], which extends the emotional cue framework in their study of gambling. However, this paper deviates from the original Card and Dahl [3] framework in a few ways that will be discussed further in the Section 2.

Another potential issue is the data used and its limitations compared to currently accessible data from the same provider. Card and Dahl [3] use IPV cases reported by the National Incident-Based Reporting Systems (NIBRS), a U.S. crime reporting database sponsored by the Federal Bureau of Investigation. Crimes are voluntarily submitted to the FBI by individual reporting agencies (usually police departments at the city and/or county level). As a result, there is considerable variation in reporting between agencies and between time periods. While the 2000 tract of NIBRS data covers less than 20% of the total U.S. population, the 2024 tract covers over 80%. The sample studied by Card and Dahl [3] only covers the period between 1995 and 2006, where the maximum percentage of the U.S. population covered by NIBRS was approximately 25%. Given the larger samples found in more recent NIBRS data, an update is likely in order.

There are other issues with the NIBRS data itself. Akiyama and Nolan [36] note the difficulty of constructing a viable dataset from the NIBRS data, which requires linking individual datasets for incident, victim, offender, arrestee, and administrative information together. NIBRS also has no individual IPV term, meaning one has to be subjectively constructed. The standard construction of IPV describes cases of aggravated assault, simple assault, or intimidation where the offender is defined as a (male) spouse (including common law and ex-spouse) or a boyfriend/girlfriend. There is also the obvious disadvantage that NIBRS only contains *reported* incidents of IPV, a crime that is often severely under-reported. Other issues with under- or misreporting have been found with NIBRS data post-Card and Dahl [3]. Bibel [37] points out that local police agency reporting can be subjective, and certain NIBRS criminal codes may function as “catch-words” that can vary across departments and states. IPV is determined through a victim–offender relationship in NIBRS; however, this is subject to subjectivity by responding officers. For example, a “stranger” or “acquaintance” assault (not IPV) may be misclassified as a “boyfriend” assault (IPV), or vice versa. Finally, Addington [38] and Chilton and Regoeczi [39] detail the nonresponse biases found in NIBRS, where nation- or state-wide changes in crime trends can be explained by the participation (or lack thereof) of certain agencies. To alleviate this problem, I employ Jacob Kaplan’s concatenated NIBRS data [40].

## 2. Data

Kaplan’s concatenated NIBRS data is considered a “cleaner” data source compared to using the raw NIBRS data outright, and as a result, has become a standard for recent studies involving NIBRS as well as recent studies involving violent crime [41,42,43]. These are used as the data source for this paper. Instead of using the 12-year, 1995–2006 sample of Card and Dahl [3], this paper uses a larger 25-year sample, from 2000 to 2024. This covers a much larger portion of the U.S. population, as well as more NFL seasons.

This larger sample allows for the inclusion of more NFL teams and more opportunities to measure unexpected results. Card and Dahl [3] examine states where only one NFL team is present to best identify supporters of one team. This is done in order to identify the highest concentration of fans of that NFL team, and therefore have the largest percentage of the population affected by an upset loss. Limitations in NIBRS participation limited this sample to only examining 8 states, and 6 NFL teams: the Carolina Panthers (South Carolina—bordering states are included with the assumption that these states should have highly concentrated supporters of a certain team), Denver Broncos (Colorado), Detroit Lions (Michigan), Kansas City Chiefs (Kansas), New England Patriots (Massachusetts, New Hampshire, and Vermont), and Tennessee Titans (Tennessee). Following this same logic, using the updated Kaplan [40] NIBRS files, this paper can expand the sample to include 20 states (plus the District of Columbia) and 16 NFL teams. These include the Arizona Cardinals (Arizona), Denver Broncos (Colorado), Washington/Redskins/Football Team/Commanders (District of Columbia and Virginia), Atlanta Falcons (Georgia), Chicago Bears (Illinois), Indianapolis Colts (Indiana), Kansas City Chiefs (Kansas), New Orleans Saints (Louisiana), New England Patriots (Massachusetts, New Hampshire, Rhode Island, and Vermont), Detroit Lions (Michigan), Minnesota Vikings (Minnesota), Las Vegas Raiders (Nevada), Carolina Panthers (North and South Carolina), Tennessee Titans (Tennessee), Seattle Seahawks (Washington), and Green Bay Packers (Wisconsin). We should expect these states to have strong fan bases toward their home team, and unexpected losses for these teams should generate the largest potential effects. There are drawbacks to this approach. For example, states with a high degree of NIBRS participation but multiple teams, such as Ohio and Texas, must be excluded. Another potential method, as followed by Arnesen and Matsuzawa [18], would be to group the geographically closest team to each agency, though this may not be perfect, as some teams (such as the Dallas Cowboys) may have a larger geographic presence than others. Also of note is that, though the entire sample runs from 2000 to 2024, not every state compiles incidents for this entire time period. That being said, every state contains at least four years of crime data.

Following Card and Dahl [3], IPV is recorded as incidents of simple assault, aggravated assault, or intimidation of a female victim committed by a male offender who was identified as the victim’s spouse, partner, or boyfriend. Following the more recent literature, such as the work of Collins [27], murders are also included. In addition, crimes committed by an ex-spouse/ex-partner/ex-boyfriend are also coded as IPV.

Following Card and Dahl [3], the sample is limited to only include Sundays during the NFL regular season (September, October, November, December, and January), when the majority of games are played. This is necessary to avoid day-of-week or seasonal variation in IPV that may skew the results. For example, Sundays see slightly higher incidents of IPV than other days. Also, following Card and Dahl [3], only crimes that occur between 12:00 pm and 12:00 am local time are included in the sample, as this period covers most NFL games. One change to the NFL schedule that has changed since the time period used for Card and Dahl [3] is the presence of European games that occur in the early morning hours. There are only a few such games in the sample, but they are removed regardless. IPV is then summed to daily totals for each agency. Following assumptions made by Arnesen and Matsuzawa [18] of the working paper of Card and Dahl [44], the sample is cut further to only include agencies that report any crime for 13 weeks of the NFL season (between 72% and 76% of the NFL season). This leaves a final sample of over 149,500 agency-day observations spread across 2789 unique, usable agencies. Summary statistics can be found in Table 1.

Unexpected NFL outcomes are determined from betting odds. Following Arnesen and Matsuzawa [18], closing point spreads are sourced from Sport Odds History. games are classified as “predicted wins”, where the local team is expected to win by more than 4 points (a point spread less than −4); “predicted losses”, where the local team is expected to lose by more than 4 points (a point spread greater than 4); and “predicted close”, where the local team is expected to either win or lose by less than 4 points (point spreads between −4 and 4). This creates a fairly balanced sample of games. Roughly 1/4th of all games are classified as “predicted win” or “predicted loss”, with approximately half of all games “predicted close”. Game outcomes come from the same site as point spreads. The key variable is upset losses: games where the local team lost but was predicted to win. Also taken into account are losses in general, losses where the game was predicted to be close, and upset wins (games where the local team won but were expected to lose). Upset wins are necessary to include as they may generate “euphoria” effects [45,46] which could reduce IPV. Any specific traits of the game (such as turnovers or penalties) are not incorporated.

Though this paper is described as an updating of Card and Dahl [3], it cannot be considered a true “replication”. Though, as a robustness check, the data will be trimmed to only include the states examined in the original paper, there are two other issues that prevent a true replication. Firstly, Card and Dahl [3] incorporate Nielsen television viewership data into some of their regressions. This data is not publicly available and therefore not included in this analysis. Secondly, Card and Dahl [3] incorporate a series of weather variables denoted as “indicators for hot, high heat index, cold, windy, rainy, and snowy days”. These are not described in detail and, as a result, are not incorporated into this paper.

## 3. Estimation

Following Card and Dahl [3] and Arnesen and Matsuzawa [18], a Poisson estimation is necessary given the relatively large number of agency-days that record zero incidents of IPV. The dependent variable is the number of IPV incidents reported by a police agency on a given Sunday during the NFL regular season. The independent variables of interest are losses for the local NFL team, upset losses (games where the local team lost but were predicted to win), “close losses” (games where the local team lost but were predicted to win), and upset wins (games where the local team won but were expected to lose). A linear approximation of the regression model is as follows:

*IPV_iws_* = *α*_0_ + *β*_1_*PredictedWin_iws_* + *β*_2_*PredictedClose_iws_* + *β*_3_*PredictedLoss_iws_* + *β*_4_*Loss_iws_* +

*β*_5_(*Loss_iws_* ∗ *PredictedWin_iws_*) + *β*_6_(*Loss_iws_* ∗ *PredictedClose_iws_*) + *β*_7_(*Win_iws_* ∗ *PredictedLoss_iws_*) + *γ*_1_*Holiday_ws_* + *δ_i_* + *ζ_w_* + *η_s_* + *ϵ_iws_*

IPV, game predictions, and game outcomes are measured for agency i, game week w, and season (year) s. The equation above represents a final estimation, though a more simplified model, only including losses, upset losses, and upset wins, is also performed. Again, using Card and Dahl [3], an indicator variable is included denoting Sundays that are also major holidays (Christmas Eve, Christmas Day, New Year’s Eve, New Year’s Day, and Halloween). Holidays and holiday weekends often see increases in IPV. Agency, game week, and season fixed effects are used to account for variation between agencies, game weeks, and seasons, respectively.

Finally, to account for agencies of different sizes beyond simple fixed effects, agency-reported population is included as an exposure variable. This approach is also employed by Arnesen and Matsuzawa [18]. However, there are some caveats to this approach. NIBRS-participating agencies correspond roughly to police departments. The jurisdictions for these agencies may often overlap (such as the city police department and the county sheriff’s department). Kaplan [47] explains that NIBRS reporting works to avoid double-counting the population. In cases where two agencies entirely overlap (for example, a college campus police department located inside a city), the non-primary agency will report a population of zero. This extends to agencies that partially overlap jurisdictions, with county police departments excluding the populations of all cities with their own police departments. As a result, agency-reported populations often undercount the true population of the agencies. To potentially reduce under-counting, all agencies with a population of zero (campus police departments, drug task forces, occasionally highway patrols, etc.) are removed from the sample.

## 4. Results

Table 2 shows the results using the full sample. Column 1 examines only losses (both predicted and unpredicted), local team losses that were predicted wins (upset losses), and upset wins (wins where the local team was expected to lose). Column 2 adds losses for games that were predicted to be close. Column 3 is the main results, following Equation (1). Cluster-robust standard errors are reported in parentheses. Fixed effects are included for game week, season, and agency, as well as an indicator for holidays, but are not shown for brevity.

The results are a departure from Card and Dahl [3]. No effect of upset losses is found. No significant relationship can be found between NFL upset losses and IPV. This result is similar to that found by Arnesen and Matsuzawa [18], which concludes that upset losses (on their own) no longer triggered spikes in IPV. Similar to Card and Dahl [3], close losses have no significant effect. Neither do pre-game expectations (wins, losses, or close games). There is a small drop in IPV corresponding to upset wins, with upset wins associated with an approximate 4% drop in IPV. This could be evidence of a “euphoria” effect, where unexpected positive results generate positive effects or reductions in harm. This is an unusual finding, as harm reduction is somewhat rare in the emotional cue literature. However, these results lose significance when predicted outcomes are incorporated into the model.

These results pose a natural question: why is the effect identified with 20-year-old data not present today? Given the presence of a considerable array of follow-up literature continuing to find evidence of “emotional cue” effects (many of which use the same NIBRS data sources), it is unlikely that the findings of Card and Dahl [3] are a fluke. A few explanations can be proposed.

One potential explanation that cannot be easily investigated is that the econometric specification described in Card and Dahl [3]—though standard at the time—is not described to the level of clarity found in economics papers of today. This leaves a few questions regarding control variables used or, more exact intricacies of Poisson estimations. In addition, using more agencies may not be as beneficial as believed, as agency controls become much harder to implement. Traditional fixed-effect Poisson models (believed to be used by Card and Dahl [3]) struggle to reach concavity when a large number of fixed effects are used due to the presence of perfect predictors. This issue is also faced by Arnesen and Matsuzawa [18], who correct for this using population as an exposure variable to reduce the total burden of fixed effects (at the cost of a reduced number of usable agencies). Out of computing necessity, this paper follows Arnesen and Matsuzawa [18], but this could be a source of the disparity. There is also the possibility of employing various other Poisson modifications, such as a zero-inflated Poisson model [48] or a (pseudo-)Poisson high-dimensional fixed-effects (HDFE) model [49]. As an additional robustness check, Column 4 of Table 2 includes a pseudo Poisson HDFE approach described by Correia et al. [49], using a similar regression framework as found in Column 3. Once again, the results are insignificant.

**Table 2:** Regression results: upset NFL losses.

Variables	IPV	IPV	IPV	IPV
	(1)	(2)	(3)	(4)
Loss	−0.0004	−0.012	−0.044	−0.039
	(0.099)	(0.013)	(0.068)	(0.062)
Loss x Predicted Win	0.030	0.027	0.044	0.038
	(0.021)	(0.021)	(0.059)	(0.057)
Loss x Predicted close		−0.009	0.028	0.027
		(0.013)	(0.065)	(0.061)
Upset Win	−0.039 *	−0.038 **	−0.071	−0.071
	(0.019)	(0.019)	(0.072)	(0.066)
Predicted Win			0.004 (0.038)	0.005 (0.037)
Predicted Close			−0.013 (0.039)	−0.010 (0.038)
Predicted Loss			0.026 (0.051)	0.034 (0.049)
Game Week FEs	YES	YES	YES	YES
Season FEs	YES	YES	YES	YES
Agency FEs	YES	YES	YES	YES
Obs.	146,657	146,657	146,657	146,657
Number of agencies	2789	2789	2789	2789

Cluster-robust standard errors in parentheses. * and ** correspond to 0.1 and 0.05 significance levels, respectively. Control variables include agency fixed effects, season fixed effects, game week fixed effects, and an indicator for holidays. Columns 1, 2, and 3 use a Poisson regression with population as an exposure variable, while Column 4 uses a Correia et al. [49] HDFE pseudo-Poisson.

A few surface-level critiques can be made as well. Given the larger number of agencies, it may be the case that IPV has increased over time. However, with this sample, the rate of IPV among agency-days stays relatively constant over the observable period. Another critique, leveled by Walker and Evans [50] in their response to Arnesen and Matsuzawa [18], is that these IPV effects are overstated because upset losses are relatively rare events. Though upset losses are—by definition—unexpected outcomes, they are not exceptionally rare. Approximately 19% of all games in the sample are upset losses for the local team, which translates to a total of 28,168 exposures, which should be plenty to register an effect. Though some seasons see a relatively small number of upsets (2002 and 2022, for example), this is counterbalanced by some seasons observing a high number of upsets (2013 and 2014).

Perhaps one of the most plausible explanations is that current NIBRS data is much more granular, and that Card and Dahl [3] identified a phenomenon unique to only a few states. To test this theory, the sample is reduced further to only include the states examined by Card and Dahl [3]: South Carolina, Colorado, Kansas, Massachusetts, New Hampshire, Vermont, and Tennessee. This smaller sample may produce results more akin to those found in the previous paper. Table 3 depicts these results. The format is similar to Table 2, with Column 1 only including losses, upset losses, and upset wins. Column 2 adds close losses, and Column 3 shows the full model. In all cases, a holiday indicator as well as agency, game week, and season fixed effects are used. Column 4 omits the state of Tennessee from the sample. Cluster-robust standard errors are reported in parentheses, and again, population is used as an exposure variable. Season, game week, and agency fixed effects are included but are not shown for brevity.

In this case, the positive relationship between upset losses and IPV is found in the simplest specification found in Column 1. Including close losses reduces the magnitude of upset losses, but it remains significant. In the specification found in Column 2, upset losses are associated with a 7.5% increase in IPV incidents. This is smaller than the standard 10% reported by Card and Dahl [3], but comparable. However, completing the full model by adding predicted outcomes renders upset losses insignificant again. Column 4 of Table 3 removes Tennessee from the sample. Tennessee sees an unusually high rate of IPV, which could be large enough to sway results [27]. Omitting Tennessee produces a positive increase in IPV from upset losses, at a magnitude of almost twice that of the amount reported by Card and Dahl [3]. However, this is significant only at the 10% level. Also unique to this sample is the presence of a “euphoria” effect. Upset wins are associated with a decrease in IPV of approximately 19.6%. A large amount, but again, this is only marginally significant. The symmetry of the result is surprising, as—because this sample is limited—an upset loss occurring on one agency day does not necessarily correlate to an upset win in another agency. While these results should be treated with a degree of caution, they serve as an excellent example of how results can be altered in the presence of a smaller geographic sample and how just one state’s inclusion or exclusion can sway results.

## 5. Conclusions

This paper examines the seminal work of Card and Dahl [3] and updates it using a larger NIBRS crime dataset. Using a Poisson estimation of agency-day IPV totals, NFL games are used as “triggers” for potential losses of control, and therefore potential spikes in IPV. Restricting the sample to only include states with one prominent NFL team nearby, and to only include Sundays during the NFL season, no significant relationship is found between upset losses and IPV. This denotes a change from the previous literature.

Though mechanical explanations for the non-effect are described in the Section 4, other psychological, social, and economic factors should be proposed as well, all of which warrant future study. One possible reason is that, given the continual growth of the NFL, it is no longer exact game outcomes that matter, but in-game effects, as demonstrated by Cardazzi et al. [16] and Mudrow [17]. With the rise in easily accessible live game statistics, in-game triggers (such as a turnover or dropped pass) could lead to spikes in IPV. Another proposal by Arnesen and Matsuzawa [18] incorporates legalized sports betting, finding no effect of upset losses on IPV for states that have not legalized sports betting, but an approximate 10% increases in IPV for upset losses in states that have legalized betting, as well as an increase in IPV following close losses in betting-legalized states, presenting a worsening emotional cue effect. Other work by Collins [27] suggests that emotional cue effects have spread beyond sports and can be found in the political process as well. It is possible that some loss-of-control-prone partners have substituted towards political triggers instead of solely sports ones.

There are other opportunities for future research. Further quantitative studies exploring the link between gender-based violence and alcohol consumption, and how both are affected by sports, are in demand. Another necessity is the study of domestic violence public service announcements (PSAs) and their effect. Though still rare, especially in the United States, a few independent organizations have timed televised anti-IPV PSAs to appear during major sporting events. These PSAs, to the author’s knowledge, have not been evaluated in a quantitative setting. Another, potentially darker explanation for the null effect found with more recent data is that loss-of-control-prone individuals have shifted to other outlets of aggression beyond IPV. These could include online aggression such as hate speech or online vandalism [51,52]. Another substitution, though, is hate crimes. Though not quite built into “emotional cue” frameworks, sports can influence hate crimes [52,53,54]. Given the increase in hate crimes in the United States [55], work exploring the link between these two under the context of emotional cues could be an important contribution. A testament to the relevance of Card and Dahl [3] is the fact that, 15 years later, it continues to spawn new research ideas and extensions, and yet there still remains room for future work.

## Figures and Tables

**Table 1:** Summary statistics.

Variable	Obs.	Mean	Std. Dev.	Min	Max
IPV	149,784	0.374	0.919	0	25
Game Spread	149,784	0.320	6.481	−26.5	17
Win	149,784	0.473	0.499	0	1
Loss	149,784	0.525	0.499	0	1
Predicted Win	149,784	0.248	0.432	0	1
Predicted Close	149,784	0.510	0.500	0	1
Predicted Loss	149,784	0.266	0.442	0	1
Upset Loss (Loss x Predicted Win)	149,784	0.188	0.391	0	1
Close Loss (Loss x Predicted Close)	149,784	0.155	0.442	0	1
Upset Win (Win x Predicted Loss)	149,784	0.055	0.228	0	1
Holidays	149,784	0.045	0.207	0	1
Year	149,784	2016.4	6.968	2000	2024
Population	149,784	62,603	112,363	31	2,721,948

**Table 3:** Regression results: only including Card–Dahl states.

Variables	IPV	IPV	IPV	IPV
	(1)	(2)	(3)	(4)
Loss	−0.083 ***	−0.008	−0.088	−0.147
	(0.016)	(0.022)	(0.097)	(0.107)
Loss x Predicted win	0.160 ***	0.075 **	0.137	0.198 *
	(0.027)	(0.030)	(0.092)	(0.102)
Loss x Predicted close		−0.014	0.073	0.132
		(0.022)	(0.095)	(0.105)
Upset Win	−0.085 ***	−0.026	−0.111	−0.196 *
	(0.031)	(0.031)	(0.103)	(0.115)
Predicted Win			−0.014	−0.060
			(0.072)	(0.082)
Predicted Close			−0.031	−0.099
			(0.071)	(0.081)
Predicted Loss			0.063	0.057
			(0.068)	(0.073)
Game Week FEs	YES	YES	YES	YES
Season FEs	YES	YES	YES	YES
Agency FEs	YES	YES	YES	YES
Obs.	58,081	58,081	58,081	50,917
Number of agencies	896	896	896	703

Standard errors in parentheses. *, **, and *** correspond to 0.1, 0.05, and 0.01 significance levels, respectively. Control variables include agency fixed effects, season fixed effects, game week fixed effects, and an indicator for holidays. Column 4 omits Tennessee.

## Data Availability

The data presented in this study are openly available in Jacob Kaplan’s Concatenated Files: National Incident-Based Reporting System (NIBRS) Data, 1991–2024 (https://doi.org/10.3886/E118281V11).
